# Anti-Inflammatory Effect of Myristicin on RAW 264.7 Macrophages Stimulated with Polyinosinic-Polycytidylic Acid

**DOI:** 10.3390/molecules16087132

**Published:** 2011-08-22

**Authors:** Ji Young Lee, Wansu Park

**Affiliations:** College of Oriental Medicine, Department of Pathology, Kyungwon University, Seongnam 461-701, Korea; Email: hangl98@naver.com

**Keywords:** myristicin, dsRNA, inflammation, macrophages, cytokine

## Abstract

Myristicin (1-allyl-5-methoxy-3,4-methylenedioxybenzene) is an active aromatic compound found in nutmeg (the seed of *Myristica fragrans*), carrot, basil, cinnamon, and parsley. Myristicin has been known to have anti-cholinergic, antibacterial, and hepatoprotective effects, however, the effects of myristicin on virus-stimulated macrophages are not fully reported. In this study, the anti-inflammatory effect of myristicin on double-stranded RNA (dsRNA)-stimulated macrophages was examined. Myristicin did not reduce the cell viability of RAW 264.7 mouse macrophages at concentrations of up to 50 µM. Myristicin significantly inhibited the production of calcium, nitric oxide (NO), interleukin (IL)-6, IL-10, interferon inducible protein-10, monocyte chemotactic protein (MCP)-1, MCP-3, granulocyte-macrophage colony-stimulating factor, macrophage inflammatory protein (MIP)-1α, MIP-1β, and leukemia inhibitory factor in dsRNA [polyinosinic-polycytidylic acid]-induced RAW 264.7 cells (*P* < 0.05). In conclusion, myristicin has anti-inflammatory properties related with its inhibition of NO, cytokines, chemokines, and growth factors in dsRNA-stimulated macrophages via the calcium pathway.

## 1. Introduction

**Figure 1:**
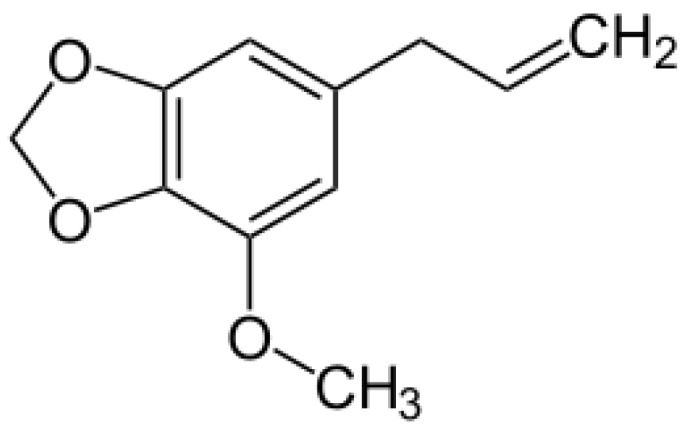
Structural formula of the alkenylbenzene myristicin.

Myristicin (1-allyl-3,4-methylenedioxy-5-methoxybenzene; CAS No. 607-91-0, Figure 1), or methoxylsafrole, is a natural aromatic alkenylbenzene constituent found in the nutmeg, the dried ripe seed of *Myristica fragrans* [1,2]. 

Myristicin is also found in basil, anise, cinnamon, clove, fennel, parsley, and star anise [3,4]. It is used as a fragrance in the cosmetic industry and as a flavouring agent in food [5]. In traditional medicine, myristicin has been used to treat cholera, stomach cramps, nausea, diarrhea, and anxiety [2]. Recently, it was reported that myristicin exerts antibacterial activity against Gram-positive and Gram-negative organisms [6], but the anti-inflammatory properties of myristicin on virus-infected macrophages are not yet fully reported.

Macrophages and their circulating monocyte form are potent defenders of physiological integrity by their mediation of crucial physiological and protective functions such as innate immunity and inflammatory reactions, foreign antigen presentation, and scavenging dead cells [7]. Nitric oxide (NO) production endows macrophages with cytotoxic activity against bacteria, viruses, fungi, protozoa, and tumor cells. However, excessive production of NO could be related with the development of septic shock, neuropathological diseases, rheumatoid arthritis, and other autoimmune disorders [8].

Cytokines, which are soluble proteins that are secreted by cells of the immune system, can alter properties of different cell types and provide essential communication signals for motile cells of the immune system [9], but excessive and uncontrolled production of these inflammatory cytokines may lead to serious systemic complications such as microcirculatory dysfunction, tissue damage, and septic shock, which can exact a high mortality [10].

The double-stranded RNA (dsRNA), which accumulates at various stages of viral replication, stimulates macrophages. Polyinosinic-polycytidylic acid (PIC) is considered as the synthetic analog of dsRNA. Like other pathogenic endotoxin, dsRNA-activated macrophages provokingly produce many kinds of inflammatory mediators, including NO, cytokines, chemokines, and growth factors, resulting in acute or chronic inflammation [11,12,13].

We have investigated the inhibitory effects of myristicin on PIC-induced inflammation using RAW 264.7 mouse macrophages and THP-1 human monocytes. In addition to the inhibitory effect on NO production in PIC-induced THP-1, our data demonstrate that myristicin inhibits the excessive production of NO, interleukin (IL)-6, IL-10, interferon inducible protein-10 (IP-10), monocyte chemotactic protein (MCP)-1, MCP-3, granulocyte-macrophage colony-stimulating factor (GM-CSF), macrophage inflammatory protein (MIP)-1α, MIP-1β, leukemia inhibitory factor (LIF), and calcium in PIC-induced RAW 264.7 macrophages. 

## 2. Results and Discussion

### 2.1. Effects of Myristicin on Cell Viability

**Figure 2:**
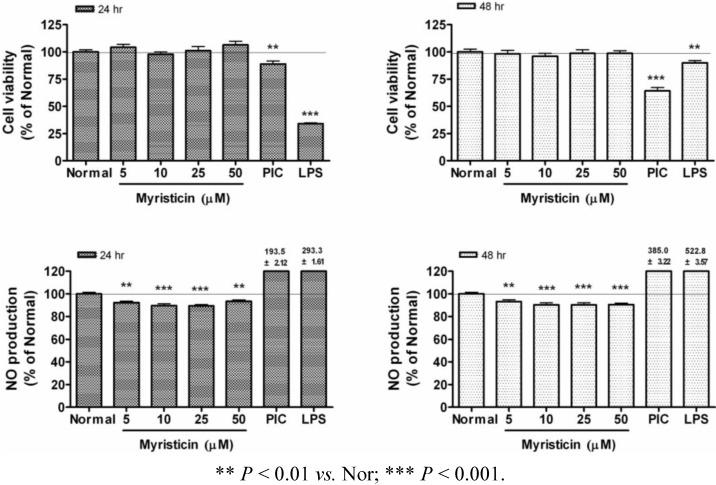
Effects of myristicin on the cell viability and NO production in RAW 264.7 mouse macrophages.After 24 and 48 h incubation with myristicin, cell viability was evaluated by a modified MTS assay and NO production was measured by the Griess reaction assay in RAW 264.7 cells. Normal group (Normal) was treated with media only. The synthetic analog of dsRNA (PIC, 50 µg/mL of polyinosinic-polycytidylic acid) and lipopolysaccharide (LPS, 1 µg/mL) were used as reference materials. Values are the mean ± SEM of three independent experiments.

Besides antibacterial activity [14], it was already reported that myristicin induces glutathione S-transferase and inhibits the tumorigenesis caused by benzo[a]pyrene in the mouse lung [15,16] Recently, it was reported that liver injury caused by lipopolysaccharides is potently prevented by myristicin treatment [17]. On the contrary, it was suggested that myristicin induces cytotoxicity in human neuroblastoma SK-N-SH cells by an apoptotic mechanism [18]. In this study, myristicin up to a concentration of 50 µM did not decrease the viability of RAW 264.7, while it decreased NO production in RAW 264.7 (Figure 2). Therefore, myristicin concentrations of up to 50 µM were chosen for subsequent experiments.

### 2.2. Effects of Myristicin on NO Production

**Figure 3:**
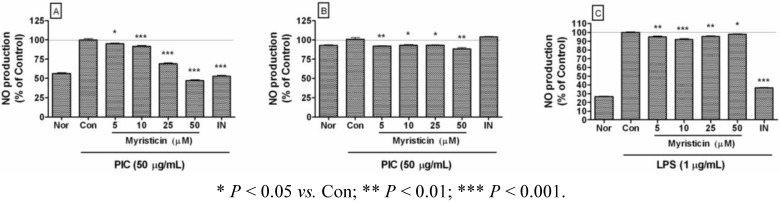
Effects of myristicin on NO production in dsRNA-stimulated RAW 264.7 mouse macrophages and THP-1 human monocytes. NO production was measured after 24 h treatment inRAW 264.7 (A, C) and THP-1 (B) by the Griess reaction assay. Normal group (Nor) was treated with media only. Control group (Con) was treated with the synthetic analog of dsRNA (PIC, 50 µg/mL of polyinosinic-polycytidylic acid) or lipopolysaccharide (LPS, 1 µg/mL) alone. IN denotes indomethacin (0.5 µM). Values are the mean ± SEM of three independent experiments.

Excess production of NO is implicated in the development of multiple organ failure, with a putative mechanism involving direct mitochondrial inhibition [19]. In this study, myristicin significantly inhibited excessive production of NO in PIC-activated RAW 264.7 (Figure 3A) and THP-1 (Figure 3B). Myristicin also inhibited excessive NO production in RAW 264.7 activated by lipopolysaccharide (LPS), a ligand of toll-like receptor 4 (Figure 3C). Since NO is believed to be a major proinflammatory mediator concerned with pathogenic infections by bacteria and viruses, these results suggest that myristicin might have anti-inflammatory activity against the pathologic and excessive production of NO in virus-stimulated macrophages and monocytes.

### 2.3. Effects of Myristicin on Cytokine Production

**Figure 4:**
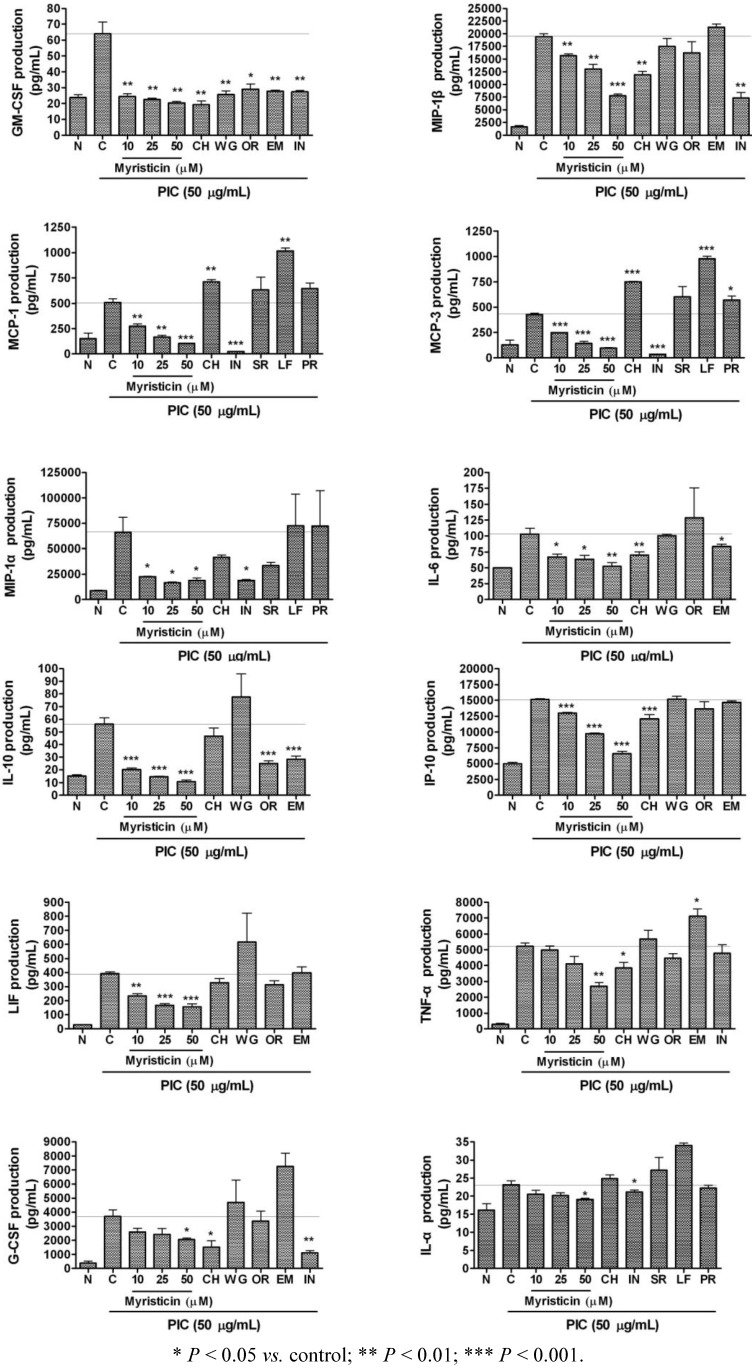
Effects of myristicin on cytokine production (IL-6, IL-10, IP-10, MCP-1, MCP-3, GM-CSF, MIP-1α, MIP-1β, LIF, TNF-α, G-CSF, and IL-1α) in dsRNA-stimulated RAW 264.7 mouse macrophages. Flourescene intensity of each cytokine in the culture medium was measured by a Multiplex bead-based cytokine assay after 24 h incubation. Normal group (N) was treated with media only. Control group (C) was treated with PIC (the synthetic analog of dsRNA, 50 µg/mL of polyinosinic-polycytidylic acid) alone. As reference materials, chrysin (CH), wogonin (WG), oroxylin A (OR), and emodin (EM) were treated at the concentration of 25 µM, while water extracts of Scutellariae Radix (SR), Lycii Fructus (LF), and Polygoni Radix (PR) were treated at 25 µg/mL. IN denotes indomethacin (0.5 µM). Values are the mean ± SEM of three independent experiments.

The effects of 24 h treatments with 10, 25, and 50 µM myristicin on cytokine production in PIC-activated RAW 264.7 macrophages are represented in Figure 4. Myristicin significantly decreased the production of IL-6, IL-10, IP-10, MCP-1, MCP-3, GM-CSF, MIP-1α, MIP-1β, and LIF in PIC-induced mouse macrophages in a dose-dependent manner. For comparing anti-inflammatory effect of myristicin with other anti-inflammatory natural materials, chrysin (CH), wogonin (WG), oroxylin A (OR), and emodin (EM) were treated at the concentration of 25 µM, while water extracts of Scutellariae Radix, Lycii Fructus, and Polygoni Radix were treated at 25 µg/mL in these experiments. Although CH, WG, OR, and EM also exerted inhibitory effects on the production of cytokines including IL-6 and GM-CSF in PIC-induced RAW 264.7, myristicin inhibited PIC-induced cytokines production more effectively than other natural materials.

It’s now recognized that resolution of inflammation is an active program controlled by temporal and spatial production of specialized chemical mediators [20]. Like other pathogenic infections, viral infection can induce excessive production of proinflammatory cytokines in immune cells, including macrophages and monocytes, resulting in acute or chronic inflammatory diseases [21].

Increased IL-6 levels often correlate with several inflammatory autoimmune diseases including Crohn’s disease, psoriasis, rheumatoid arthritis, systemic-onset juvenile chronic arthritis, osteoporosis, polyclonal plasmacytosis, malignant plasmacytoma, and encephalomyelitis [20]. In addition it has been demonstrated that IL-6 and soluble form of IL-6 receptor are increased in both serum and intestinal tissues of the patients with active Crohn's disease [22]. LIF, a member of IL-6-family, was also reported to be increased in the colonic mucosa of patients with inflammatory bowel disease and play a critical role in the susceptibility of colonic host cells to tumor growth in ulcerative colitis patients [23]. IL-10 is a pleiotropic cytokine that modulates the function of adaptive immune-related cells. IL-10, although traditionally considered an anti-inflammatory cytokine, has also been implicated in promoting abnormal angiogenesis in the eye and in the pathobiology of autoimmune diseases such as lupus and encephalomyelitis [24]. In the present study, myristicin inhibited excessive production of IL-6, LIF, and IL-10 in PIC-induced RAW 264.7 macrophages. These results support the contention that myristicin may regulate chronic autoimmune diseases such as Crohn’s disease, psoriasis and encephalomyelitis, which can be provoked with viral infection.

Inflammation is a central feature of many lung diseases, including pneumonia, asthma, cystic fibrosis, chronic bronchitis, and emphysema [25]. Adcock and Caramori [25] have reported that asthma, a chronic inflammatory disease of the airway characterized by cellular infiltration and activation, might be induced by overexpression of chemokines and cytokines (such as GM-CSF, MIP-1α, and MIP-1β) released on exposure to inhaled allergens. During lung inflammation, it has been also reported that MCP-1, IP-10, and GM-CSF were increased in the bronchoalveolar lavage fluid [26,27]. MCP-1 was also reported to be hypersecreted in airway hyperreactivity and chronic airway inflammatory diseases such as asthma [28]. Increased expression of MCP-3 mRNA has been reported in the airway submucosa of patients with asthma [29]. In this study, myristicin displayed significant inhibitory effects on PIC-induced MCP-1, MCP-3, GM-CSF, MIP-1α, and MIP-1β production in RAW 264.7 cells. The current results suggest that myristicin may relieve pulmonary inflammatory diseases such as acute lung injury, bronchial pneumonia, and chronic asthma concerned with viral infection. But myristicin, only at the concentration of 50 µM, significantly inhibited the production of some cytokines such as TNF-α, G-CSF, and IL-1α in PIC-induced RAW 264.7 cells. Thus, myristicin is suggested to be applied in higher concentrations for evaluation of anti-inflammatory effects concerned with TNF-α, G-CSF, and IL-1α.

### 2.4. Effects of Myristicin on Intracellular Calcium Production

Pathogenic oxidative stress with infection results in macrophage reprogramming with a transient increase of intracellular calcium via the lipid membrane dissociation of the calcium-bound protein annexin VI. This increased cytosolic calcium, in turn, results in the activation of calcium-dependent kinases, leading to enhanced proinflammatory activation [30]. In the start of the inflammation cascade, the endoplasmic reticulum (ER) calcium stores are reduced and intracellular calcium concentration is increased, resulting in ER stress-mediated transcription factor activation and increased inflammatory gene expression [31].

**Figure 5:**
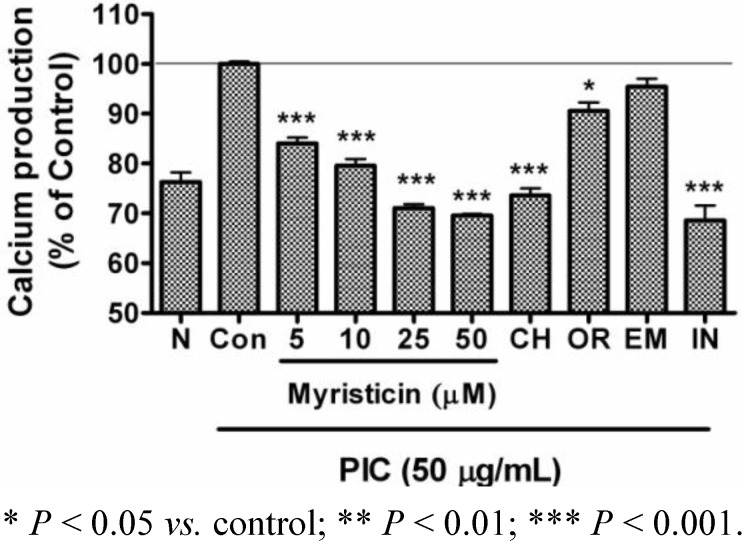
Effects of myristicin on calcium production in dsRNA-stimulated RAW 264.7 mouse macrophages. Intracellular production of calcium after 24 h treatment was measured with Fluo-4 calcium assay. Normal group (N) was treated with media only. Control group (Con) was treated with PIC (the synthetic analog of dsRNA, 50 µg/mL of polyinosinic-polycytidylic acid) alone. As reference materials, chrysin (CH), oroxylin A (OR), and emodin (EM) were treated at the concentration of 25 µM. IN denotes indomethacin (0.5 µM). Values are the mean ± SEM of three independent experiments.

In the present study, myristicin reduced the increase of intracellular calcium level in PIC-activated RAW 264.7 macrophages (Figure 5). Thus, it can be suggested that myristicin down-regulates excessive production of inflammatory mediators in PIC-induced macrophages via the calcium pathway. But whether the intracellular calcium concentration is increased via the calcium-bound membrane protein mobilization or release of calcium from ER could not be confirmed in this study.

## 3. Experimental

### 3.1. Reagents

Dulbecco’s Modified Eagle’s Medium (DMEM), RPMI-1640 medium (RPMI), heat-inactivated fetal bovine serum (FBS), penicillin, streptomycin, phosphate-buffered saline (PBS, pH 7.4), and other tissue culture reagents were purchased from Gibco BRL (Grand Island, NY, USA). PIC, griess reagent, and all other chemicals were purchased from Sigma-Aldrich (St. Louis, MO, USA). CellTiter 96 Aqueous one solution cell proliferation assay kit was purchased from Promega (Madison, WI, USA). The multiplex bead-based cytokine assay kits used for the determination of cytokine concentration were purchased from Bio-Rad (Hercules, CA, USA), Panomics (Redwood City, CA, USA), and Millipore (Billerica, MA, USA). The Fluo-4 calcium assay kit was purchased from Molecular Probes (Eugene, OR, USA).

### 3.2. Cell Culture and Viability

RAW 264.7 mouse macrophages and THP-1 human monocytes were obtained from the Korea Cell Line Bank (Seoul, Korea). RAW 264.7 were cultured in DMEM, as THP-1 in RPMI, supplemented with 10% FBS containing 100 U/mL of penicillin and 100 µg/mL of streptomycin at 37 °C in a 5% CO_2_ humidified incubator. Cell viability was assessed by a modified MTS assay using CellTiter 96 Aqueous one solution cell proliferation assay kit (Promega) [32].

### 3.3. Quantification of NO Production

NO concentration in culture medium was determined by the Griess reaction assay [33]. Specifically, 100 µL of supernatant from each well was mixed with 100 µL of Griess reagent in wells of a 96-well plate. After an incubation of 15 minutes at room temperature, the optical density was determined at 540 nm with a microplate reader (Bio-Rad).

### 3.4. Multiplex Bead-Based Cytokine Assay

Cytokines released from treated RAW 264.7 macrophages were measured in cell culture supernatants using a Luminex assay based on xMAP technology. This assay was performed with Bio-Plex cytokine assay kits (Bio-Rad), Procarta cytokine assay kits (Panomics), Milliplex kits (Millipore) and Bio-Plex 200 suspension array system (Bio-Rad) as described previously [33,34]. Standard curves for each cytokine were generated using the kit-supplied reference cytokine samples. Production of the following cytokines was assessed: IL-6, IL-10, IP-10, MCP-1, MCP-3, GM-CSF, MIP-1α, MIP-1β, LIF, TNF-α, IL-1α, and G-CSF.

### 3.5. Intracellular Calcium Assay

After RAW 264.7 cells were seeded in wells of 96-well plates, PIC and myristicin were added to the culture medium, and incubation was carried out for 24 h at 37 °C. Thereafter, the medium was removed and cells were incubated with 100 µL of the Fluo-4 dye loading solution for 30 minutes at 37 °C. After incubation, the fluorescence intensity of each well was determined spectrofluorometrically (Dynex, West Sussex, UK) with excitation and emission filters of 485 nm and 535 nm, respectively.

### 3.6. Statistical Analysis

The results shown are summarized from three independent experiments and represent the mean ± SEM. Significant differences were examined using a Student’s *t*-test with SPSS 11.0 software (SPSS, Chicago, IL, USA).

## 4. Conclusions

Although the precise mechanisms regulating the anti-inflammatory activity of myristicin are not yet known, the current study demonstrates that myristicin has anti-inflammatory properties related with its inhibition of NO, IL-6, IL-10, IP-10, MCP-1, MCP-3, GM-CSF, MIP-1α, MIP-1β, and LIF in PIC-stimulated macrophages via the calcium pathway.
